# Development of a behavior change intervention to improve physical activity in patients with COPD using the behavior change wheel: a non-randomized trial

**DOI:** 10.1038/s41598-023-50099-z

**Published:** 2023-12-21

**Authors:** Xinyue Xiang, Maomao Han, Xiaolin Luo, Yudi Yu, Xiaorong Lu, Shasha Cai, Lihua Huang

**Affiliations:** 1 Department of Nursing, The First Affiliated Hospital, Zhejiang University School of Medicine, Hangzhou, Zhejiang Province China; 2 Department of Nursing, Haining People’s Hospital, Haining, Zhejiang Province China; 3 Zhejiang Evaluation Center for Medical Service and Administration, Hangzhou, Zhejiang Province China

**Keywords:** Respiratory tract diseases, Clinical trial design

## Abstract

The aim of this study was to evaluate whether a theory-based behavior change intervention could promote changes in physical activity (PA) and sedentary behavior (SB) among patients with chronic obstructive pulmonary disease (COPD), as well as its effects on symptoms of dyspnea, lung function, exercise capacity, self-efficacy, and health-related quality of life (HRQoL). A quasi-experimental design and convenience sampling were adopted. A total of 92 patients with stable COPD were recruited from outpatient and inpatient centers of two hospitals in Zhejiang Province, China. Both the experimental and control groups received standard medical care provided in the hospital. The experimental group performed a PA program based on the behavior change wheel theory. Outcomes were measured at baseline (T0) and after 4 weeks (T1), 8 weeks (T2), and 12 weeks of the intervention (T3). The primary outcome was PA measured by the International Physical Activity Questionnaire (IPAQ). Secondary outcomes included SB measured by the IPAQ, dyspnea measured by the modified Medical Research Council (mMRC) questionnaire, exercise capacity assessed by 6-min walk distance (6MWD), self-efficacy measured by the Exercise Self-Regulatory Efficacy Scale (EX-SRES), and HRQoL measured by the COPD Assessment Test (CAT). In addition, we measured lung function using a spirometer at baseline and 12 weeks. Of the 89 patients included in this study, 64 were male (71.91%), with a mean age of 67.03 ± 6.15 years. At 12 weeks, the improvements in PA, SB, mMRC, 6MWD, EX-SRES and CAT were all statistically significant (*P* < 0.05) in the experimental group compared to the control group. Repeated measures analysis of variance showed that there were group effects and time effects on total PA, SB, mMRC, 6MWD, EX-SRES, and CAT in both groups (*P* < 0.001). However, there was no significant difference in pulmonary function between the two groups before and after intervention (*P* < 0.05). The PA program based on theory significantly increased PA levels, reduced sedentary time, enhanced exercise capacity and self-efficacy as well as HRQoL in patients with stable COPD. Due to the limited intervention time in this study, the pulmonary function of COPD patients may not be reversed in a short time, and the long-term effect of this program on the pulmonary function of patients needs to be further explored.

**Trial registration:** Clinical Trials.gov (ChiCTR2200060590). Registered 05/06/2022.

## Introduction

Chronic obstructive pulmonary disease (COPD) is a common, preventable and treatable disease characterized by persistent and progressive restrictions on air circulation^1^. Over 90% of people with COPD suffer from dyspnea, which often limits their ability to perform daily activities, including physical activity (PA)^2,3^. Physical inactivity is a risk factor for premature mortality and several noncommunicable diseases^4^. Compared to healthy persons of the same age, the amount, intensity and duration of PA in patients with COPD is significantly reduced^5^. The 2021 Global Initiative for Chronic Obstructive Pulmonary Disease (GOLD) guidelines recommend that all patients with COPD maintain and increase their PA, irrespective of disease severity. However, PA levels for most COPD patients are not ideal and cannot meet the criteria recommended in the guidelines^6^. Current data suggest that patients with COPD reduce their physical activity early in the course of the disease^7,8^. In addition, maintaining PA is very difficult for COPD patients, mainly due to disease specificity, including dyspnea, fatigue, pain, comorbidities, and skeletal muscle dysfunction^9,10^. Physical inactivity is a strong predictor of all-cause mortality in COPD patients and is associated with increased healthcare utilization and worse health-related quality of life (HRQoL)^11,12^. Therefore, improving PA in patients with COPD is of great significance for the prognosis of the disease.

Pulmonary rehabilitation (PR) is an essential component of COPD management and has been proven to improve the physical and psychological ability of patients^13,14^. Once PR is completed, the benefits gained begin to decline unless patients continue to exercise regularly. Furthermore, the enhancement of exercise tolerance after PR cannot be converted into an improvement in PA^15^. According to the consensus of PR, to maintain the long-term effects of exercise interventions, health behavior change is crucial.Reviews of the current evidence report that there is insufficient evidence for improvement in PA with strategies including exercise training, PA counseling and pharmacological management. The optimal timing, components, duration and models for interventions are still unclear^16^. Physical activity is a complex behavior, which is affected by individual physiological, psychological, social environment and other factors. It is difficult to promote the change of physical activity behavior in COPD patients^17^. Behavior change intervention based on theory can provide rigorous, systematic and feasible scientific guidance for the design of intervention programs so that the intervention has long-term effectiveness^18^. To help researchers understand the mechanism of individual behavior, the behavior change wheel (BCW) ^19^ was developed from 19 behavior change frameworks. It consists of three layers, in which the core is the COM-B (‘capability’, ‘opportunity’, ‘motivation’ and ‘behavior’) model. This framework provides a systematic and standardized development process, starting from the behavioral analysis of the problem, understanding the intrinsic mechanism of behavior generation, and selecting the best intervention program^19^. Currently, BCW has been widely used in the design of many interventions, including health-related behaviors such as smoking cessation^20^, healthy diet^21^, physical activity^22^, and reduction of alcohol intake^23^. In addition, foreign scholars have applied BCW to reduce sedentary behavior of COPD patients^24^, enhance exacerbation-related self-management^25^, develop a home-based rehabilitation mHealth system and promote the improvement of COPD patients' exercise ability^26^. The application of BCW theory in COPD patients in China remains to be further studied.

Therefore, the aim of this study was to develop a PA intervention for stable COPD patients in China based on the BCW and to evaluate the feasibility and effectiveness of the method in the hope of providing guidance for the treatment of such patients, with the results reported as follows.

## Methods

### Study design

This study adopted a quasi-experiment design (nonequivalent control group and non-synchronized, pretest–posttest design). Due to it is difficult to achieve double-blind intervention in this study, non-randomized controlled trials were selected. We used a convenient sampling method to select COPD patients in the outpatient and ward of the Department of Respiratory Medicine of the First Affiliated Hospital of Zhejiang University and Haining People's Hospital. In order to better control the confounding variables, this study used a coin toss method for inpatients and outpatients. The patients in the first ward of respiratory medicine were taken as the experimental group, and the patients in the second ward of respiratory medicine were taken as the control group. The study was approved by the Institutional Research Committee of the First Affiliated Hospital of Zhejiang University and registered on ClinicalTrials.gov (ChiCTR2200060590). All participants provided written informed consent, and all ethical considerations were observed according to the Helsinki Declaration.

### Participants

Participants were recruited from May 2022 to July 2022. Inclusion criteria were as follows: (1) the diagnosis of stable COPD patients according to the GOLD (2022) guidelines, with a postbronchodilator ratio of the forced expiratory volume in one second (FEV1) to the forced vital capacity (FVC) < 0.70, and absence of exacerbation in the 4 weeks before collection^1^; (2) patients with stable vital signs, can cooperate to complete the 6MWT; and (3 competent to provide informed consent. The exclusion criteria were as follows: (1) acute exacerbation of COPD according to GOLD; (2) patients with severe heart, kidney, liver, muscle, and other systemic diseases; (3) patients with bronchial asthma, pulmonary bullae, pulmonary hypertension, severe pneumonia, malignant tumors and other serious lung diseases; (4) patients with life-threatening conditions during exercise, such as severe pulmonary hypertension, exercise-induced syncope, unstable angina, and recent myocardial infarction; (5) limb dysfunction or severe joint pain; and (6) patients with severe mental disorders and cognitive impairment. Exclusion and drop-out criteria: (1) patients who did not meet the inclusion criteria were identified after enrollment; (2) patients with poor compliance resulting in incomplete data collection.

### Sample size calculation

We calculated sample size based on the assumption that patients who received a behavior-change intervention improved the primary outcome (physical activity levle). Sample size was calculated using the formula: n1 = n2 = (Zα + Zβ)2 * 2σ2/δ2. The two-group sample size ratio was 1:1, statistical power of 90%, and a 2-sided significance level of 0.05. According to the pilot study, σ = 210.56 and δ = 154.08 were substituted into the formula. Allowing for an 15% loss to follow-up, we planned to recruit 92 participants (46 per group).

### Intervention

**Figure 1:**
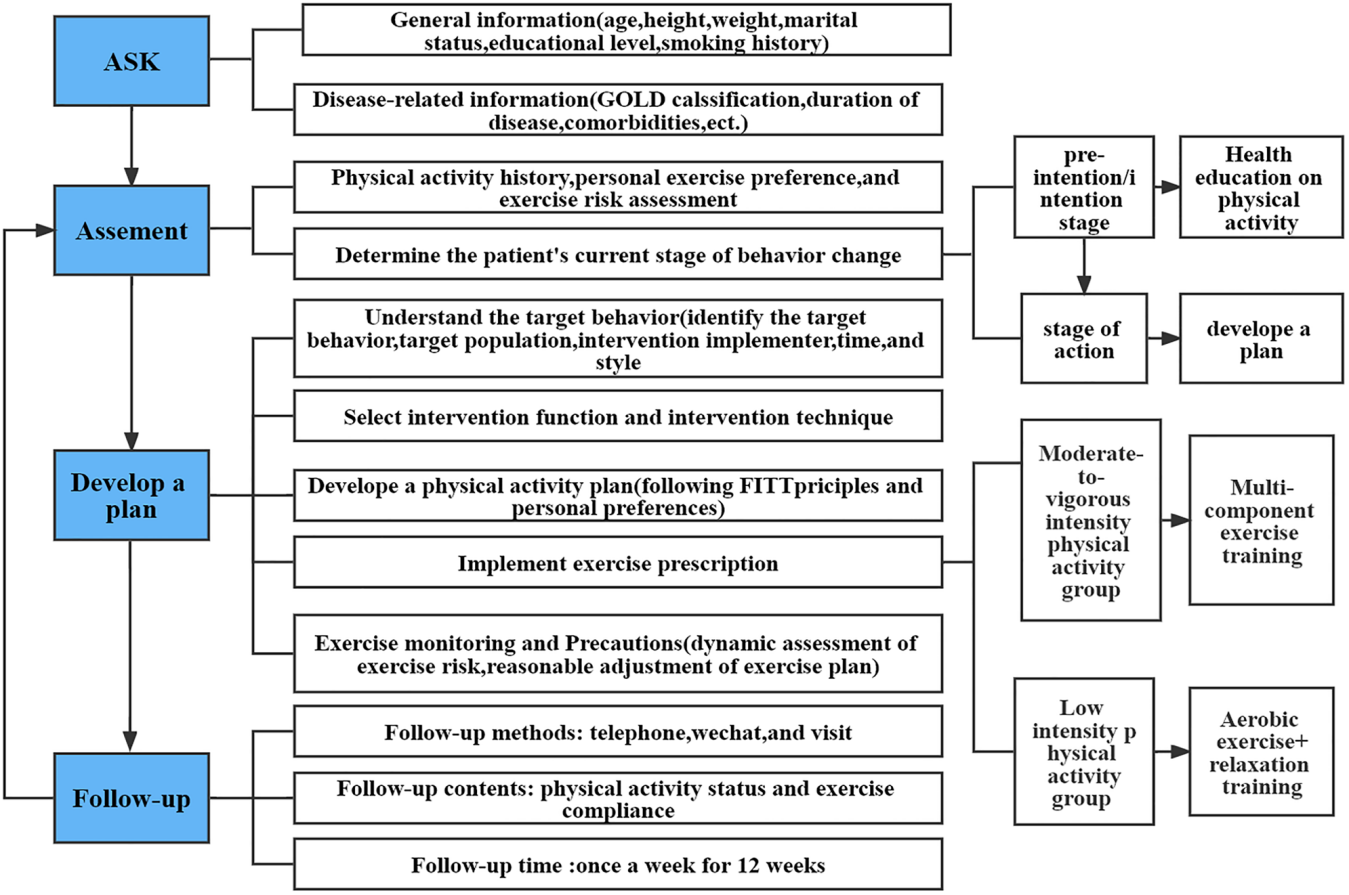
Flowchart of the intervention for the protocol.

This study is mainly divided into two groups, experimental group and control group. Both the experimental and control groups received standard medical care provided in the hospital. The control group was given routine intervention and health education guidance, including basic knowledge of the disease, smoking cessation, self-management skills, an action plan for COPD acute exacerbations, exercise training guidance (respiratory training), social psychological guidance and nutritional support. After discharge, telephone follow-up was conducted once a week for 15–20 min for 12 weeks. The main contents of follow-up included asking patients about their current health status, informing patients to seek medical treatment in time if their condition worsened, and answering patients' common health questions. In the experimental group, a PA program based on BCW theory was implemented on the basis of routine nursing, as follows. A flowchart of the intervention for the protocol is provided in Fig. 1.

#### Building the intervention team

A nurse-led multidisciplinary team was established before the intervention. There were 13 members in the group, including 1 director of the nursing department, 2 head nurses, 2 chief physicians of respiratory medicine, 1 rehabilitation therapist, 4 nurses of respiratory medicine and 3 postgraduates of nursing. Among them, the director of the nursing department was responsible for the overall control and quality control of the research to ensure the rigor and rationality of the research; the head nurse was responsible for personnel arrangement and organization of training; respiratory physicians were responsible for the supervision and guidance of research programs; the rehabilitation therapist was responsible for prephysical activity assessment and safety monitoring during exercise; respiratory nurses were responsible for the implementation of intervention programs, health education and follow-up; and master’s students were mainly responsible for literature retrieval and quality evaluation, construction of scheme and data collection.

#### Develop a physical activity plan

At the beginning of this phase, a literature review and expert consultation were used to formulate the components and steps of the intervention. Then, combined with the disease demands of COPD patients in China, we initially constructed the first draft of a PA intervention program for COPD patients under the guidance of BCW theory. The full scoping review has been published previously^17^. Then, our team invited experts in the field of COPD (8 experts from the field of respiratory, chronic disease management and exercise rehabilitation, all of whom were associate professors or above; bachelor’s degree or above) to analyze and discuss the PA intervention program and, combined with clinical practice experience, put forward suggestions for modification. The modified PA program was applied to 12 patients for a pretest to verify the clinical applicability and operability of the program. Finally, a PA intervention for COPD patients based on BCW theory was developed. The detailed development process is shown in Additional file 2.

#### Implementing the intervention

1. Motivational interview

On the first day of admission, the nurse introduced the ward environment and the purpose of the study to the patient and her family, obtained the patient’s consent and cooperation, and signed the informed consent form. The patient’s medical history, PA status and other information related to the disease were inquired. Subsequently, patients were provided with customized health education manuals, including the epidemiology, risk factors, disease symptoms and clinical manifestations of COPD. We assessed the stages of PA behavior change in COPD patients according to the Transtheoretical Model model (TTM). TTM stages include Precontemplation, Contemplation, Preparation, Action, Maintenance. TTM is a successful framework for guiding behavior change programs for several health behaviors. The type, duration, and intensity of the PA should be geared towards which stage the individual finds themselves in at the time. One-on-one motivational interview was used to assess the patients' current behavior change stage: in the pre-intention/intention stage, the health education of PA knowledge should be continued to be strengthened; in the preparation/action phase, nurses should help patients develop an action plan and provide information support.

2. Assess physical activity

At the study visit, we used the International Physical Activity Questionnaire (IPAQ) to assess the past and current PA history of patients, including the type, intensity, time and frequency of activity, and to understand the daily activity level of the patients; introduce the definition of PA and SB, the benefits of PA to health and the dangers of prolonged sedentary behavior to patients and their families, correct their incorrect perception, and establish a correct concept of disease. In addition, we should pay more attention to the psychological nursing of patients, understand the possible concerns of patients and their families in activities, strengthen psychological counseling for their bad emotions, and guide patients to master the skills to relieve psychological stress, such as listening to soothing music, reasonable exercise, mindfulness meditation practice, and planting green flowers.

2. Make a plan

According to the PA assessment results and lung function status at baseline, COPD patients were divided into a moderate- and vigorous-intensity group and a low-intensity group. Individual PA levels were classified according to criteria developed by the IPAQ group (see Additional file 1). Both groups also needed to meet the requirements of resting blood pressure < 140/90 mmHg and normal rise of blood pressure during exercise; recommence exercise SpO_2_ > 90%. According to the FITT (frequency, intensity, time and type) principles, combined with patients' own conditions, individualized exercise prescriptions were made for patients (see Table 1).

**Table 1:** Exercise prescription.

Intervention time	Frequency	Type		Duration time (min)	Intensity
1–2 weeks after discharge	3–5 times/week	Warm-up	Upper and lower limb stretching exercises	5–10	Intensity of the exercise was monitored by heart rate reserve and Borg rating of perceived exertion (RPE) scale
		Aerobic exercise	walk	10 ~ 15	
		resistance training	Elastic band exercises	10 (2sets, repeat 10–15 times)	
		Relaxation training	extension exercises	5	
3–4 weeks after discharge	3–5 times/week	warm-up	upper and lower limb stretching exercises	5–10	
		Aerobic exercise	walk	15–20	
		resistance training	Elastic band exercises	10 (2sets, repeat 10–15 times)	
		Relaxation training	extension exercises	5	
6–8 weeks after discharge	3–5 times/week	warm-up	upper and lower limb stretching exercises	5–10	
		aerobic exercise	walk	20–25	
		resistance training	Elastic band exercises	15 (3sets, repeat 8–12 times)	
		Relaxation training	extension exercises	5	
8–12 weeks after discharge	3–5 times/week	warm-up	upper and lower limb stretching exercises	5–10	
		Aerobic exercise	walk	20–25	
		resistance training	Elastic band exercises	15 (3sets, repeat 8–12 times)	
		Relaxation training	extension exercises	5

4. Set a goal

(I) Short-term goal: patients in the low-PA group might start with low-intensity PA (for example, walking at a leisurely pace, riding a bike on flat ground and lifting light weights) and gradually increase the intensity of exercise; patients in the moderate- to high-intensity PA group recommended performing moderate-intensity exercise (brisk walking) at least 5 days a week. (II) Long-term goal: All patients’ daily walking time increased, sedentary time decreased, dyspnea improved, and cardiopulmonary function and quality of life improved.

5. Implement exercise prescription

In the first week after discharge, exercise guidance was provided by the exercise rehabilitation therapist, and the patients in the moderate and vigorous PA groups received comprehensive training combined with aerobic exercise and resistance exercise. Patients in the low PA group only performed aerobic exercise; both groups performed warm-up and relaxation training for 5–10 min before and after exercise. Walking was recommended as the main training method of aerobic exercise. The workout session should last between 25 and 60 min with a frequency of 3–5 times every week. Resistance exercise is recommended to use an elastic band, mainly for the upper limbs, including three movements: standing front horizontal lift, arm bend lift in the standing position and chest push in the sitting position. Each movement lasts approximately 3 s, repeated 10–15 times as a set, for a total of 2 sets. The interval between the sessions was 2 min and 3 times a week.

6 Exercise monitoring and safety precautions

(I) Explain the safety precautions related to the activities to the patients and their families in detail. Stretching and relaxation training can be performed before and after exercise. The duration was 5–10 min, and a slight stretching sensation was dominant. If the patient is over 75 years old and has poor physical fitness, it is best to be accompanied by a family member. (II) Identify the signs of termination of exercise: once a patient has chest pain, dyspnea (modified Borg dyspnea scale > 5 points), dizziness, nausea and vomiting, pallor, sweating, resting heart rate above 120 beats per minute, systolic blood pressure ≥ 180 mmHg and/or diastolic blood pressure > 100 mmHg (1 mmHg = 0.133 kPa), SpO_2_ < 85%, workout should be stopped immediately; exercise risk assessment should be performed before PA recovery, and SpO2 should be higher than 90% when restarting exercise. During the activity, if limited by personal factors or environmental conditions, intermittent exercise can be carried out, but not for more than 2 days. (III) Intensity of exercise can be determined by heart rate reserve combined with subjective fatigue score. The maximum heart rate = (220−age), the target heart rate = (maximum heart rate-resting heart rate) × (60–70%) + resting heart rate, or using the RPE scale (12–14 points). (IV) Guide patients to use a WeChat pedometer for self-monitoring of daily steps; use an activity log to record daily activity, including duration, intensity, and activity experience, to understand their progress or meet the target.

7. Regular follow-up

After discharge, the patients were followed up by telephone or WeChat (that is a smartphone application) every week for 15–20 min for 12 weeks. The main contents of follow-up included asking the patient about the activity history of the past week, assessing whether the patient's exercise goal had been completed, reminding the patient to continue exercising, recording the results on the follow-up form, and giving feedback in a timely manner.

### Assessments

#### General data

The questionnaire was designed by researchers on the basis of reviewing the relevant literature. (I) Demographic data, including age, sex, weight, marital status, education level, smoking status, etc.; (II) disease-related data, including GOLD classification, year of COPD diagnosis, comorbidities, etc.

#### International physical activity questionnaire, IPAQ

The questionnaire was developed by the World Health Organization in 1998. It includes both short and long versions and is recognized as one of the valid assessment questionnaires for measuring PA levels^27^. In this study, a long volume questionnaire was used to investigate the physical activities of the subjects in the past 7 days. The questionnaire included 27 items in total, including 4 types of physical activities, including occupation, housework, transportation and leisure, and 5 types of sedentary activities. PA was expressed as metabolic equivalent (MET). The metabolic equivalent of tasks was calculated using the frequency and duration of physical activity and was reported as MET-min/week.

#### Pulmonary function tests

Forced vital capacity (FVC), forced expiratory volume in one second (FEV1), and the FEV1/FVC ratio were measured by a computerized spirometer. The spirometer was calibrated before use to ensure that it worked properly. Before the examination, we understood the patient’s medical history and treatment in detail, determined whether the indication for spirometry was met, and paid attention to exclude relevant contraindications. Introduce the procedure and movement to the patient and guide the patient to practice to master the movement more quickly. The patient takes a sitting position with his chest straight, feet relaxed and the head naturally level or slightly tilted. According to the test curve and indicators, patients were guided to perform measurement procedures and corresponding actions. The measurement is repeated, and the best result is selected.

#### Modified Medical Research Council scale (mMRC)

The mMRC scale is a self-rating tool to measure the degree of disability that breathlessness poses on day-to-day activities on a scale from 0 (no breathlessness except on strenuous exercise) to 4 (too breathless to leave the house or breathless when dressing or undressing)^28^.

#### Six minutes walking test, 6MWT

The 6MWT was undertaken in accordance with the American Thoracic Society (ATS)/European Respiratory Society (ERS) guidelines. It was carried out by the team members in the corridor of the respiratory ward of the hospital, with a length of 30 m and red warning lines marked at both ends of the ground. Blood oxygen saturation and heart rate were monitored during the experiment using a portable oximeter. At the end of the experiment, walking distance was recorded, and the Borg scale was used to assess dyspnea and fatigue.

#### Exercise Self-Regulatory Efficacy Scale, EX-SRES

This scale was developed by Davis et al. and can be used as an effective tool to assess exercise efficacy beliefs in COPD patients^29^. Translated into Chinese by Yu-Han Tsai^30^ et al., the scale has 16 items, which reflect the confidence of COPD patients to continue to exercise under the conditions of bad weather, pain, exercise alone, busyness, no support from others, hypoxia, and fatigue. Each item is measured with 0–10 points, where 0 points means “no confidence” and 10 points means “very confident”. The total score is the sum of the scores of 16 items, and a total score < 53 is classified as low-level exercise self-efficacy. The total scores ranged from 53 to 106, which were classified as moderate exercise self-efficacy. A total score > 106 was classified as high exercise self-efficacy, and the higher the score was, the higher the confidence that COPD patients could adhere to exercise. The Chinese version of the instrument had an overall Cronbach’s alpha of 0.925.

#### COPD assessment test, CAT

The scale was developed by Jones PW and can be used to assess the clinical symptoms and quality of life of patients^31^. Eight items were included: cough, expectoration, chest tightness, mobility, ability of daily living, ability to go out, sleep, and energy. Each item was scored from 0 to 5, and the total score was from 0 to 40, with higher scores indicating more severe illness and lower quality of life. A score of 0–10 indicates that quality of life is slightly affected, 11–20 indicates that quality of life is moderately affected, 21–30 indicates that quality of life is severely affected, and 30–40 indicates that quality of life is extremely severely affected. The Cronbach’s α coefficient of the scale was 0.82.

#### Data collection

The general data and outcome indicators of this study were collected by two trained and qualified evaluators. The collection time of each indicator was as follows: (I) Baseline data: collected on the day of admission; (II) The outcome indicators included the main outcome indicators: physical activity, which was collected on the day of admission (T0), week 4 (T1), week 8 (T2) and week 12 (T3). Secondary outcome indicators: sedentary time, exercise ability, mMRC, exercise self-efficacy and CAT were collected at T0, T1, T2 and T3. Pulmonary function indexes were measured at T0 and T3. In addition to the baseline data collected in the hospital, the outcome indicators at the T1, T2 and T3 time points were mainly collected by telephone or WeChat. Electronic questionnaires were distributed to patients and filled in by the patients themselves. Questionnaires were collected on site. Vague questions or uncertain options should be checked with the patient before filling them in.

#### Data analysis

All data were input into Excel 2017. After double-checking and verification, a database was established, and SPSS 26.0 statistical software was used for statistical analysis. To evaluate the normality of the data, the Shapiro–Wilk test was utilized. Data are presented as the mean ± standard deviation (SD) for continuous variables and frequencies and percentages for categorical variables. We used the difference-in-difference (DID) approach to evaluate the impact of our intervention because it allowed us to make comparisons before and after exposure to our intervention between the experimental and control groups. Two independent sample *t* tests were used for comparisons between groups, and paired sample *t* tests were used for comparisons within groups. We used chi-squared tests to evaluate associations among categorical variables.

Repeated measures analysis of variance (ANOVA) was used to evaluate the change trend of the scale scores at different time points. The effect size of repeated measures ANOVA was calculated as partial et squared ($\eta^{2}$) and classified as small 0.01, medium 0.06 and large 0.14. Pairwise comparisons were conducted using the Bonferroni method. Intraclass correlation coefficients were calculated to quantify the magnitude of the between-group differences in quantitative variables, thereby confirming the practical significance of our findings. Intra-class correlation coefficient (ICC) is used to measure the consistency of repeated measures. A *P* value < 0.05 was considered to indicate statistical significance.

## Results

### General information

**Figure 2:**
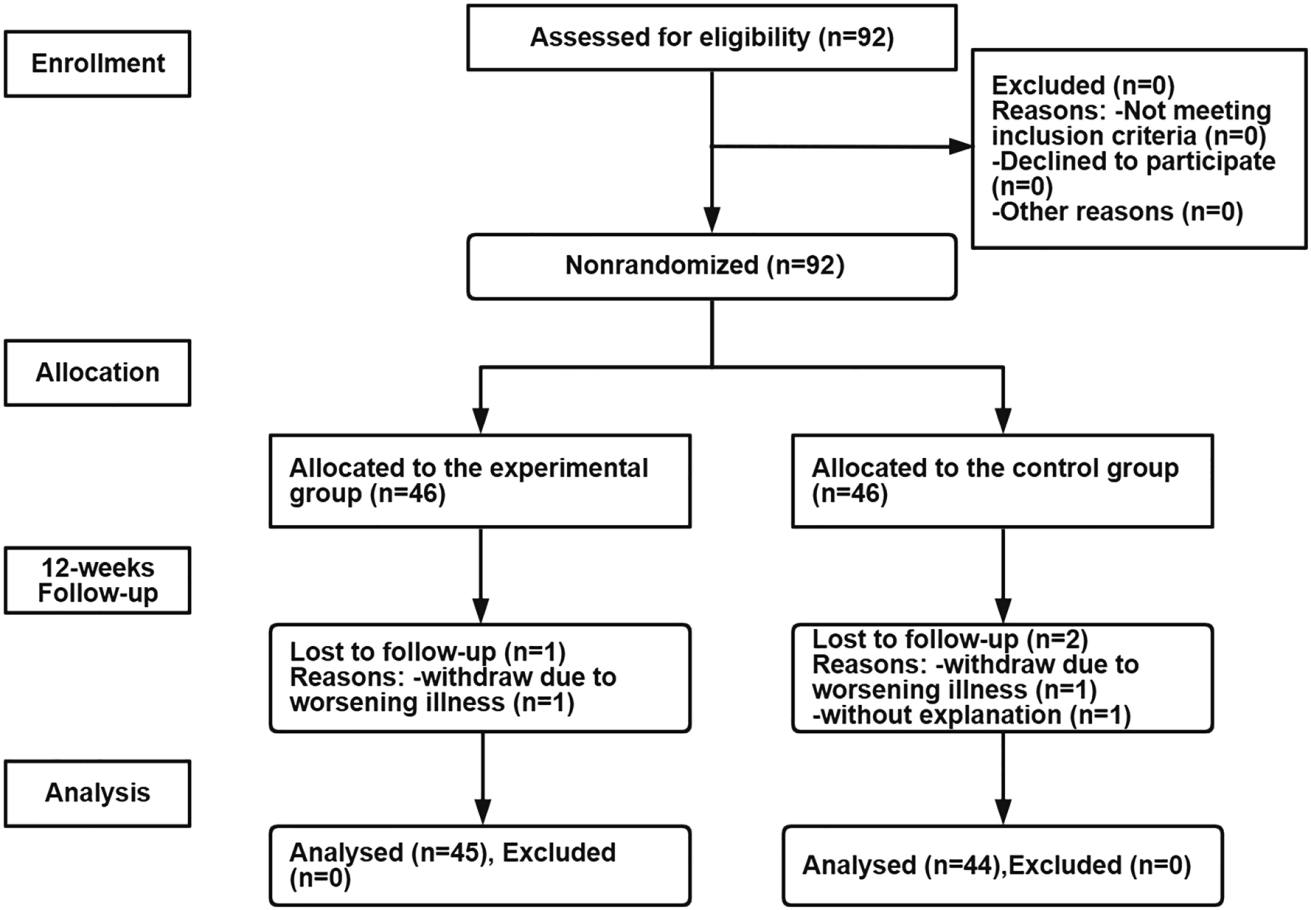
Study flowchart.

We had a total of 89 participants, where 44 participants in the control group and 45 participants in the experimental group completed the baseline survey (Fig. 2). The demographic and clinical characteristics of the study population are shown in Table 1. Of the 89 patients included in the analyses, 64 were male (71.91%), with a mean age of 67.03 ± 6.15 years. Most of them (66.29%) had an education level of primary school or below, and 87.64% were retired. The duration of disease was mainly 5–10 years, accounting for 51.69%. Among the enrolled patients, there were 14 (15.7%) patients with GOLD stage I, 42 (47.2%) patients with GOLD stage II, and 33 (37.1%) patients with GOLD stage II. There was no statistically significant difference between the experimental and control groups at baseline in terms of basic demographic characteristics (*P* > 0.05 for all) (Table 2).

**Table 2:** Characteristics of participants (N = 89).

Characteristics	Control group (n = 44)	Experimental group (n = 45)	*P* value
Gender			
Male	32 (72.72%)	32 (71.11%)	0.865^b^
Female	12 (27.27%)	13 (28.89%)	
Age (years)	67.39 ± 6.61	66.69 ± 5.71	0.595^a^
BMI (kg/m^2^)	22.55 ± 2.49	22.99 ± 3.03	0.458^a^
Education level			
Primary school and below	28 (63.63%)	31 (68.89%)	0.952^b^
Middle school	11 (25.00%)	9 (20.00%)	
High school	3 (6.82%)	3 (6.67%)	
College graduate or beyond	2 (4.55%)	2 (4.44%)	
Work status			
Full-time employment	5 (11.36%)	6 (13.33%)	0.778^b^
Retirement	39 (88.64%)	39 (86.67%)	
Marital status			
Single	6 (4.55%)	5 (6.67%)	0.717^b^
Married/widowed	38 (95.45%)	40 (93.33%)	
Living conditions			
Live alone	5 (11.36%)	7 (15.56%)	0.563^b^
Live with others	39 (88.64%)	38 (84.44%)	
Smoking status			
Never smoker	11 (25.00%)	8 (17.78%)	0.650^b^
Former smoker	15 (34.09%)	15 (33.33%)	
Current smoker	18 (40.91%)	22 (48.89%)	
Years of diagnosis COPD			
< 1 year	6 (13.64%)	5 (11.11%)	0.881^b^
1–5 years	7 (15.91%)	9 (20.00%)	
5–10 years	22 (50.00%)	24 (53.33%)	
> 10 years	9 (20.45%)	7 (15.56%)	
GOLD classification			
I (mild)	8 (18.18%)	6 (13.33%)	0.384^b^
II (moderate)	18 (40.91%)	25 (55.56%)	
III (severe)	18 (40.91%)	14 (31.11%)	
Comorbidities			
Heart disease	25 (56.81%)	22 (48.89%)	0.503^b^
Diabetes	7 (15.91%)	12 (26.67%)	
Hypertension	6 (13.64%)	6 (13.33%)	
Others	6 (13.64%)	5 (11.11%)	

Continuous values are presented as the mean ± standard deviation, and categorical variables are presented as numbers (percentage).

BMI, body mass index; kg, kilograms; GOLD, Global Initiative for Chronic Obstructive Lung Disease.

^a^Independent t test.

^b^Chi-squared test.

### Comparison of the outcome variables between the two groups before and after the intervention

**Figure 3:**
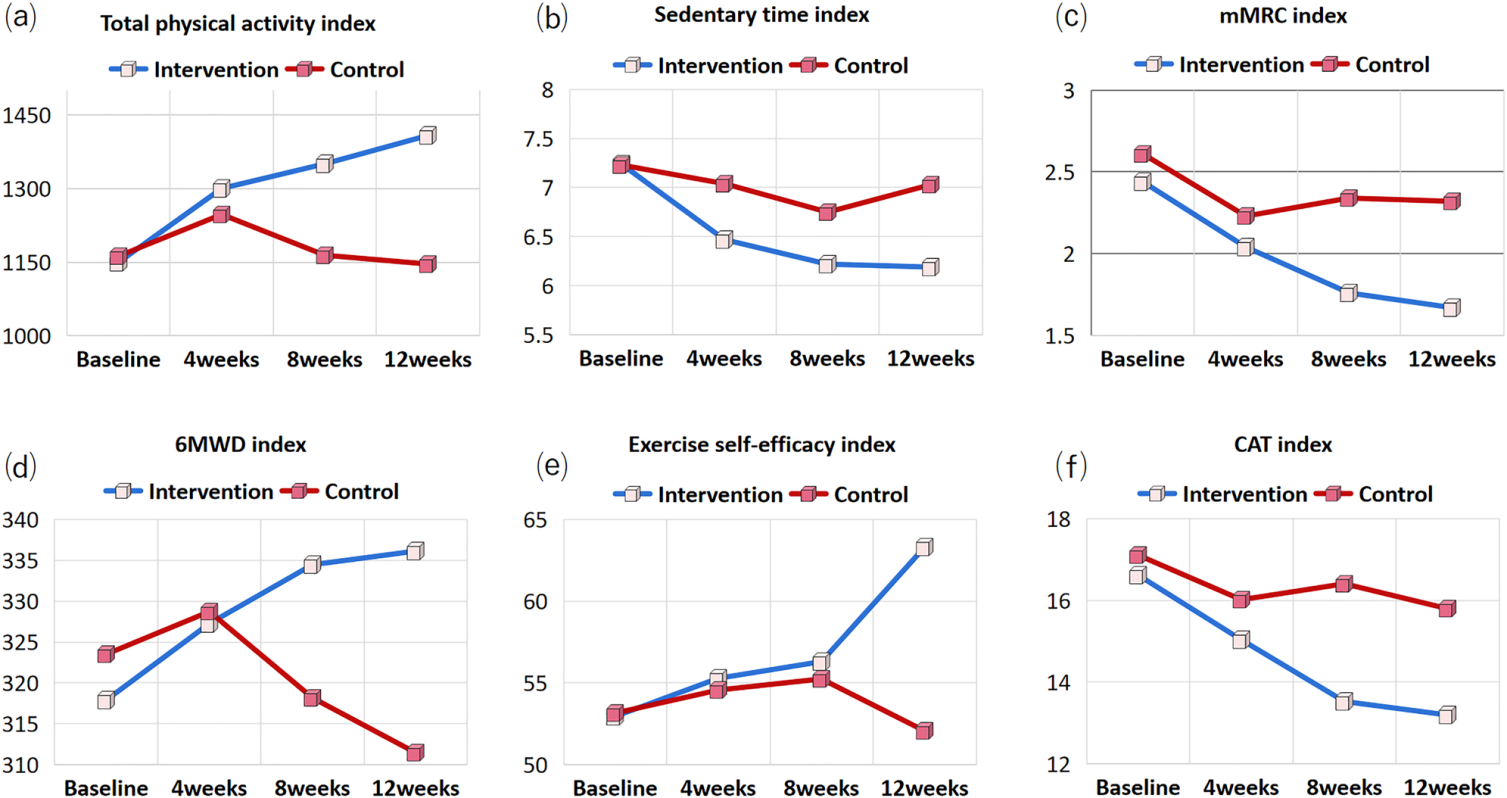
Changes in outcomes (**a**) total physical activity, (**b**) sedentary time (**c**), mMRC, (**d**) 6MWD, (**e**) exercise efficacy, and (**f**) CAT over time in the experimental and control groups.

The primary analyses showed that the PA intervention program provided a significant benefit over usual care (Table 3) (see additional file 1). At baseline, there was no significant difference in PA level between the two groups (*t* = 0.138, *P* = 0.891). After the intervention, the PA level of the experimental group increased significantly from the 4th week. It reached statistical significance only at 8 weeks and persisted throughout the study period (Table 3, Fig. 3). Patients in the experimental group showed significant improvements at 12 weeks compared with baseline in total PA (ICC = 0.64, 95CI [0.50, 0.75]), SB (ICC = 0.82, 95CI [0.71, 0.89]), mMRC (ICC = 0.71, 95CI [0.60, 0.80]), and self-efficacy (ICC = 0.31, 95CI [0.06, 0.51) (*P* < 0.05), whereas no such trend was found in the control group (Table 3). Furthermore, compared with baseline, the 6MWT (ICC = 0.74, 95CI [0.62, 0.82]) and CAT (ICC = 0.63, 95CI [0.46, 0.75]) score of the two groups were improved, but the improvement effect of the experimental group was more obvious, and the difference between the two groups was statistically significant after intervention (*P* < 0.05). The difference in FEV1% between the experimental group and the control group before and after intervention was statistically significant (DID-4.45, 95% CI [− 6.62, − 2.28]), *P* < 0.001) (Table 4).

**Table 3:** Comparison of the outcome variables between the two groups before and after the intervention.

Variables	Group	Baseline (T0)	4 weeks (T1)	8 weeks (T2)	12 weeks (T3)	*F*1(*p*)	${\eta }^{2}$	*F*2(*p*)	${\eta }^{2}$	*F*3(*p*)	${\eta }^{2}$
PA	Exp	1150.02 ± 434.79	1300.40 ± 387.17 ^a^	1350.67 ± 347.34 ^a^	1408.44 ± 361.78 ^a^	4.433 (**0.038**)	0.048	5.155 (**0.003**)	0.154	1.886 (0.138)	0.062
	Con	1162.50 ± 420.87	1247.45 ± 485.85	1164.20 ± 387.17	1146.32 ± 321.55						
	t	0.138	–0.573	–2.393	–3.610						
	p	0.891	0.568	**0.019**	**0.001**						
SB	Exp	7.24 ± 0.92	6.47 ± 0.83 ^a^	6.22 ± 0.54^ab^	6.19 ± 0.52 ^abc^	10.714 (**0.002**)	0.110	23.030 (**< 0.001**)	0.448	14.817(**< 0.001**)	0.343
	Con	7.23 ± 0.10	7.04 ± 0.96	6.75 ± 0.72	7.03 ± 0.90						
	t	-0.052	2.980	3.872	5.356						
	p	0.959	**0.004**	** < 0.001**	** < 0.001**						
mMRC	Exp	2.44 ± 1.06	2.04 ± 1.15	1.76 ± 0.91 ^a^	1.67 ± 0.91 ^ac^	6.893 (**0.010**)	0.073	7.899 (**< 0.001**)	0.83	1.874 (0.138)	0.021
	Con	2.61 ± 1.10	2.32 ± 0.98 ^a^	2.34 ± 1.08 ^a^	2.32 ± 1.03						
	t	1.135	1.521	3.430	3.442						
	p	0.259	0.132	**0.001**	**0.001**						
Exercise self-efficacy	Exp	52.96 ± 5.42	55.29 ± 5.43 ^a^	56.31 ± 5.45 ^a^	63.31 ± 8.10 ^abc^	22.282 (**< 0.001**)	0.204	10.840 (**< 0.001**)	0.277	17.274 (**< 0.001**)	0.379
	Con	53.16 ± 4.76	54.57 ± 4.26 ^a^	55.25 ± 5.41 ^a^	52.07 ± 4.52 ^bc^						
	t	0.188	-0.703	-0.922	0.359						
	p	0.851	0.484	0.359	** < 0.001**						
CAT	Exp	16.62 ± 2.75	15.04 ± 2.02 ^a^	13.53 ± 1.55 ^ab^	13.20 ± 1.91 ^ab^	29.976 (**< 0.001**)	0.256	20.336 (**< 0.001**)	0.418	4.441 (**< 0.001**)	0.136
	Con	17.11 ± 2.79	16.02 ± 2.14 ^a^	16.41 ± 2.18	15.80 ± 1.96 ^ac^						
	t	0.837	2.217	7.188	6.321						
	p	0.405	**0.029**	** < 0.001**	** < 0.001**						
6MWD	Exp	317.91 ± 15.10	327.27 ± 15.04 ^a^	324.47 ± 12.05 ^ab^	336.13 ± 11.98 ^ab^	13.465 (**< 0.001**)	0.134	14.875 (**< 0.001**)	0.344	30.545 (**< 0.001**)	0.519
	Con	323.52 ± 14.89	328.18 ± 10.68 ^a^	318.18 ± 110.68 ^ab^	311.48 ± 11.41 ^abc^						
	t	1.765	0.457	–6.743	–9.938						
	p	0.081	0.649	** < 0.001**	** < 0.001**						

Significant valuesare in [bold].

Exp , Experimental group; Con, Control group; PA, physical activity; SB, sedentary behavior; mMRC, modified Medical Research Council dyspnea scale; 6MWD, 6-min walking distance; CAT , COPD Assessment Test; *F*1,= Group effect; *F*2, Time effect; F3, Interaction effect.

*P value of the intervention group compared with the control group was calculated by repeated measurement (T1-T3).

^a^Compared with baseline *P* < 0.05.

^b^Compared with 4 weeks after treatment, *P* < 0.05. c Compared with 8 weeks after treatment, P < 0.05.

**Table 4:** Difference-in-differences of lung function.

Variables	Experimental group (*n* = 45)			Control group (*n* = 44)			DID between groups (95% CI)	*P* value
	Baseline	12 weeks	Within-group effect (*P*)	Baseline	12 weeks	Within-group effect (*P*)		
FEV1%	57.18 ± 15.87	60.58 ± 15.31	**0.01**	57.84 ± 16.62	56.77 ± 14.30	0.12	− 4.45 (− 6.62 to − 2.28)	** < 0.001**
FEV1	1.61 ± 0.35	1.69 ± 0.34	0.23	1.58 ± 0.60	1.59 ± 0.34	0.96	− 0.11 (− 0.34 to 0.13)	0.37
FVC	2.78 ± 0.56	2.76 ± 0.58	0.37	2.67 ± 0.92	2.76 ± 0.55	0.61	− 0.07 (− 0.46 to 0.33)	0.74
FEV1/FVC	58.03 ± 6.94	59.12 ± 6.75	0.13	58.98 ± 6.71	57.88 ± 6.94	0.43	− 2.40 (− 6.14 to 1.33)	0.21

Significant values are in [bold].

FEV1, forced expiratory volume in 1 s; FVC, forced vital capacity; DID, difference-in-difference.

Results of one-way repeated-measures ANOVA showed that the main effect of the scores on PA (F = 4.433, $\eta^{2}$ = 0.048, P < 0.05), SB (F = 10.714, $\eta^{2}$ = 0.110, P < 0.05), mMRC (F = 6.893, $\eta^{2}$ = 0.073, P < 0.01), Exercise self-efficacy (F = 22.282, $\eta^{2}$ = 0.204, P < 0.001), CAT (F = 29.976, $\eta^{2}$ = 0.256, P < 0.001) and 6MWD (F = 13.465, $\eta^{2}$ = 0.134, P < 0.001) from baseline to 3 months follow-up was significant (all *P* < 0.05). This indicates that the PA behavior change intervention program is effective (see Table 3).

Meanwhile, the interaction effects was significant for SB (*F* = 30.545, $\eta^{2}$ = 0.343, *P* < 0.001), 6MWD (*F* = 30.545, $\eta^{2}$ = 0.519, *P* < 0.001),exercise self-efficacy (*F* = 17.274, $\eta^{2}$ = 0.379, *P* < 0.001), and CAT scores(*F* = 4.441, $\eta^{2}$ = 0.136, *P* < 0.05) (Table 3, Fig. 3), indicating that these outcomes of participants were affected by the time and intervention. In addition, although the interaction effect was not significantly different in the PA and mMRC, the independent samples t test results showed that the PA level increased and the mMRC score decreased significantly more in the experimental group than in the control group at 8 and 12 weeks of intervention (Table 3) (see additional file 1).

The results of pairwise comparison showed that SB scores were lower than baseline levels at 4 weeks, 8 weeks, and 12 weeks of intervention (*P* < 0.05). The scores of exercise self-efficacy were higher than the baseline level at 4 weeks, 8 weeks and 12 weeks of intervention (*P* < 0.05). The 6MWD score was higher than the baseline level at 4 weeks and 8 weeks of intervention, and the CAT score was lower than the baseline level at 4 weeks and 8 weeks.

Figure 3 shows the trend in the scores of each outcome index in the two groups. The total PA, 6MWD, and exercise self-efficacy scores of the experimental group increased significantly over time, and reached the best level at week 12. However, the curve of the control group did not change much after the intervention, showing a downward trend. The sedentary time, mMRC score, and CAT score of the experimental group significantly decreased, while the score of the control group showed an increasing trend.

## Discussion

To the best of our knowledge, this is the first study to evaluate the effect of a PA program based on BCW theory on PA levels, sedentary time, dyspnea, exercise capacity, lung function and HRQoL among patients with COPD. The results showed that, compared to the control group, the PA of patients in the experimental group increased significantly and the sedentary time decreased after 12 weeks of intervention. The program increased the patient's exercise capacity and self-efficacy, and improved the patient's health-related quality of life. Due to the limited intervention time in this study, the pulmonary function of COPD patients may not be reversed in a short time, and the long-term effect of this program on the pulmonary function of patients needs to be further explored.

PA and prolonged sitting are strongly associated with increased all-cause mortality in patients with COPD^32^. Therefore, improving patients' PA levels and reducing SB are critical for disease management in patients with COPD. Our results showed that there were significant differences in total PA and SB scores between the two groups at 12 weeks after intervention (*P* < 0.05); the total PA score of the experimental group was significantly higher than that of the control group, and the sedentary time score was lower than that of the control group. Repeated measures analysis of variance showed that with the extension of the intervention time, the sedentary time of the two groups improved. However, the effect of the experimental group was more obvious than that of the control group. Our results revealed that theory-based behavior change intervention programs can help to better understand the mechanisms of behavior change and promote more durable health behavior change^33^. Wotton et al. explored the effect of ground walking training on PA and sedentary time of COPD patients, and the results showed that there was no statistically significant difference in PA and sedentary time between the two groups^31^. The reasons may be as follows: PA and SB are complex behaviors that are affected by individual, physiological, psychological, social environment and other comprehensive factors and behavioral change. Behavioral changes cannot be achieved by relying on exercise training alone^34^. Therefore, based on the guidance of the theoretical framework, our study improved patients' knowledge and ability related to PA through motivational interviews, setting behavioral goals, making exercise plans, strengthening self-feedback and monitoring, and regular follow-up to stimulate patients' intrinsic motivation and fundamentally establish healthy behaviors. On the other hand, to ensure the effectiveness of exercise training, the construction of this study protocol is based on relevant guidelines, expert consensus and literature, combined with expert opinions and preliminary experimental results, which fully considers the disease characteristics and personal preferences of domestic COPD patients and has strong operability. Although COPD patients experience many barriers to PA, an in-depth understanding of the patient’s PA experience through one-on-one interviews can help patients overcome these barriers. Multicomponent activities, including aerobic exercise, resistance training and relaxation training, can maximize the patient's exercise capacity and muscle strength. Moreover, the training method is simple and easy, with moderate intensity, which is easier for COPD patients to accept and adhere to. It is beneficial to improve patients’ participation and compliance and encourage patients to develop good exercise habits.

Lung function is of great significance for monitoring disease progression in COPD patients. FEV1/FVC is a sensitive index to evaluate airflow limitation. Due to the complexity and long duration of COPD, lung function is impaired to varying degrees, leading to dyspnea and decreased exercise tolerance^13^. Therefore, delaying the progression of lung function and improving the quality of life of patients are the main goals of nursing intervention and the urgent needs of COPD patients. Our results showed that the behavior change intervention improved some pulmonary function measures in COPD patients, which was consistent with previous studies^35^. Chen et al., implemented a PR intervention based on the transtheoretical model for COPD patients for 9 months. The results showed that the pulmonary function of patients in the experimental group was better than that in the control group after the intervention^36^. The reasons may be that the subjects of our study were mainly elderly patients, and the intervention time was only 3 months. COPD is a relatively slow progressive disease, and the mechanism of lung function improvement is complex, so it is difficult to make substantial changes through short-term intervention. A systematic review showed that the longer the duration of exercise rehabilitation exercise, typically 12–18 months, the greater the health benefits that patients can achieve^37^. Although the current recommended pulmonary rehabilitation program is 6–8 weeks, intensive intervention can help achieve more significant clinical effects in patients who may need a longer time to achieve clinical improvement^38^. This suggests that we need to extend the intervention and follow-up time to further evaluate the sustained effect of the program on lung function changes in COPD patients to provide practical evidence for clinical practice.

Shortness of breath is one of the most common symptoms of COPD patients and a major predictor of hospitalization and mortality^39^. COPD patients often avoid activities due to dyspnea symptoms and thus become more sedentary, leading to skeletal muscle adaptation, social isolation and negative emotions, which will further reduce patients’ functional ability and quality of life^40^. The results of this study demonstrated that patients in both groups had high dyspnea scores before the intervention. After 12 weeks of intervention, the dyspnea score of the experimental group was significantly lower than that of the control group, which was consistent with the findings of Mahler and Mendes^41,42^, suggesting that the PA intervention had a significant effect on improving the symptoms of dyspnea in COPD patients. A study by Troosters showed that although behavioral intervention combined with exercise rehabilitation training did not increase the level of physical activity of patients, it reduced activity-related dyspnea^43^. Based on BCW theory, our study deeply analyzed the barriers and promoting factors of PA in patients from the three levels of motivation, ability and opportunity of individual behavior. The motivational communication method and the “5A” model were used to implement intervention management, which corrected the catastrophic understanding of dyspnea of patients, reduced the fear of PA, and strengthened the understanding of the importance of exercise. It satisfies the dynamic needs of patients and improves the enthusiasm and confidence of patients to participate in the exercise rehabilitation program. Second, exercise rehabilitation helps to reduce patients' negative emotions, such as anxiety and depression, as well as self-reported activity-related dyspnea, achieving long-term beneficial health outcomes^44^.

The 6MWT is an important marker of disease severity in patients with COPD, which has been shown to be related to lung function and HRQoL and can predict patient mortality^45^. HRQOL is a subjective feeling that describes an individual's own living condition from physical (physiological), psychological and social aspects. For COPD patients, the improvement of exercise capacity and the change in adaptive behavior are the premise of improving the health status of patients. Regular exercise training increases cardiopulmonary fitness, in part due to increased mitochondrial density and oxidase activity. Our study showed that the theory-based behavior change intervention program was effective in improving the exercise capacity and quality of life of patients, which was similar to the results of domestic and foreign studies. Breyer et al. showed that after three months of intervention, Nordic walking had a positive impact on exercise capacity and quality of life of COPD patients compared to the control group, and the intervention effect lasted for 9 months^46^. Chen et al.^47^ implemented a pedometal-based PA intervention for COPD patients, and the results showed that compared with before intervention, the daily step number of patients in the pedometer group increased from 4768.4 ± 2643.3 to 7042.7 ± 4281.9 steps after 6 weeks of intervention, and the CAT score decreased from 14.9 ± 8.8 points to 11.5 ± 7.5 points (*P* = 0.03), confirming that PA intervention can effectively improve patients' exercise ability and quality of life. We developed a practical exercise program through multidisciplinary collaboration (including respiratory medicine, rehabilitation medicine, and behavioral science) to continuously enhance patients' understanding of exercise rehabilitation and PA as a lifestyle throughout the life span. A telephone follow-up was arranged once a week after discharge, and the rehabilitation therapists conducted exercise risk assessments. The exercise intensity was adjusted according to the patient's symptoms and exercise ability in time, which ensured the safety and effectiveness of the exercise, thus avoiding the occurrence of exercise-related adverse events. Exercise intervention can not only improve the patient's exercise ability and reduce the level of systemic inflammation but also promote mental health and quality of life.

Low exercise self-efficacy in COPD patients was associated with low PA, self-reported health status, and exercise ability. The results of this study showed that there were statistically significant differences in the group effect, time effect and interaction effect of exercise self-efficacy between the two groups (*P* < 0.05), indicating that with the extension of intervention time, the exercise self-efficacy of the two groups would be improved, and the effect of the experimental group was more significant than that of the control group. At 12 weeks after intervention, there was a statistically significant difference in the scores of exercise self-efficacy between the two groups (*P* < 0.05), which confirmed the positive impact of this behavior change intervention program on patients' self-efficacy, consistent with the findings of the Robinson study^48^. The reasons were as follows: (I) Given the large heterogeneity of COPD patients, not all patients can benefit from the same treatment or intervention. Therefore, the assessment of exercise self-efficacy at baseline and individualized physical activity counseling in this study were more helpful to help patients with low self-efficacy achieve or maintain physical activity goals. (II) To ensure patient safety, the responsible nurse will explain exercise-related knowledge to patients and issue health education manuals. The exercise guidance video was pushed through WeChat to correct the wrong training position and improve the confidence of patients to adhere to the exercise. (III) Exercise training was supervised by rehabilitation therapists, followed the physical activity guidelines for COPD patients, formulated exercise prescriptions, helped patients cope with movement disorders, and improved their confidence in coping with movement disorders. (IV) Strengthening social support (from family, friends and medical staff), as well as successful personal experience sharing, were the most effective ways to enhance patients' exercise self-efficacy.

### Limitations

Considering the factors of manpower, material resources and time, there are still some limitations in this study. First, compared with experimental studies, quasi-experimental studies are more feasible and practical in population intervention research. There are several limitations associated with quasi-experimental designs, which include lack of randomization, selection bias, performance bias and reporting bias. Large sample, multi-center, randomized controlled trials can be further carried out in the future. Second, we measured PA using a questionnaire but not using an accelerometer. Questionnaire-based surveys may be affected by recall bias, resulting in higher self-reported levels of PA. To obtain more accurate data, objective measurement tools such as accelerometers could be used to assess physical activity levels in the future. Third, the intervention and follow-up time of the project was only 12 weeks, which was relatively short. Exercise is a long-term process, and the health benefits of patients may be more significant with the increase in the duration of the intervention period. Therefore, we will continue to explore the lasting effects of this behavior change program on PA in COPD patients.

## Conclusions

In summary, our results showed that the BCW theory-based PA program increased PA levels, reduced sedentary time, enhanced exercise capacity and self-efficacy, and improved some lung function measures as well as HRQoL in patients with stable COPD. In the future, we can further explore the continuous effect of this program on lung function changes in COPD patients.

### Supplementary Information

Supplementary Information 1.

Supplementary Information 2.

## Data Availability

Data from this study are available on request from the corresponding author (lihuahuang818@zju.edu.cn).

## Abbreviations

PA: Physical activity
COPD: Chronic obstructive pulmonary disease
SB: Sedentary behavior
HRQoL: Health-related quality of life
BCW: Behavior Change Wheel
mMRC: Modified Medical Research Council
6MWT: Six-minute walking test
GOLD: Global Initiative for Chronic Obstructive Lung Disease
PR: Pulmonary rehabilitation
FEV1: Forced expiratory volume in 1 s
FVC: Forced vital capacity
