# Supplementary material for: Development of a behavior change intervention to improve physical activity in patients with COPD using the behavior change wheel: a non-randomized trial
Source: Sci Rep. 2023 Dec 21;13:22929. doi: 10.1038/s41598-023-50099-z (PMC10739839; doi:10.1038/s41598-023-50099-z)

**Additional file 1. Detailed and additional data of this manuscript**

**Table 1.**

Exercise prescription.

| **Intervention time** | **Frequency** | **Type** | | **Duration time (min)** | **Intensity** |
| --- | --- | --- | --- | --- | --- |
| 1~2w after discharge | 3~5 times /week | warm-up | upper and lower limb stretching exercises | 5~10 | Intensity of the exercise was monitored by heart rate reserve and Borg's Rating of Perceived Exertion (RPE) scale |
|  |  | aerobic exercise | walk | 10~15 |  |
|  |  | resistance training | elastic band exercises | 10 (2sets, repeat 10-15 times) |  |
|  |  | relaxation training | extension exercises | 5 |  |
| 3~4w after discharge | 3~5 times /week | warm-up | upper and lower limb stretching exercises | 5~10 |  |
|  |  | aerobic exercise | walk | 15~20 |  |
|  |  | resistance training | elastic band exercises | 10 (2sets, repeat 10-15 times) |  |
|  |  | relaxation training | extension exercises | 5 |  |
| 6~8w after discharge | 3~5 times /week | warm-up | upper and lower limb stretching exercises | 5~10 |  |
|  |  | aerobic exercise | walk | 20~25 |  |
|  |  | resistance training | elastic band exercises | 15 (3sets, repeat 8-12 times) |  |
|  |  | relaxation training | extension exercises | 5 |  |
| 8~12w after discharge | 3~5 times /week | warm-up | upper and lower limb stretching exercises | 5~10 |  |
|  |  | aerobic exercise | walk | 20~25 |  |
|  |  | resistance training | elastic band exercises | 15 (3sets, repeat 8-12 times) |  |
|  |  | relaxation training | extension exercises | 5 |  |

**Table 2**

Characteristics of participants (N=89).

| Characteristics | Control group(n=44) | Experimental group (n=45) | *P-*value |
| --- | --- | --- | --- |
| Gender | | | |
| Male | 32(72.72%) | 32(71.11%) | 0.865^b^ |
| Female | 12(27.27%) | 13(28.89%) |  |
| Age(years) | 67.39±6.61 | 66.69±5.71 | 0.595^a^ |
| BMI(kg/m^2^) | 22.55±2.49 | 22.99±3.03 | 0.458^a^ |
| Education level | | | |
| Primary school and below | 28(63.63%) | 31(68.89%) | 0.952^b^ |
| Middle school | 11(25.00%) | 9(20.00%) |  |
| High school | 3(6.82%) | 3(6.67%) |  |
| College graduate or beyond | 2(4.55%) | 2(4.44%) |  |
| Work status | | | |
| Full-time employment | 5(11.36%) | 6(13.33%) | 0.778^b^ |
| Retirement | 39(88.64%) | 39(86.67%) |  |
| Marital status | | | |
| Single | 6(4.55%) | 5(6.67%) | 0.717^b^ |
| Married/widowed | 38(95.45%) | 40(93.33%) |  |
| Living conditions | | | |
| Live alone | 5(11.36%) | 7(15.56%) | 0.563^b^ |
| Live with others | 39(88.64%) | 38(84.44%) |  |
| Smoking status | | | |
| Never smoker | 11(25.00%) | 8(17.78%) | 0.650^b^ |
| Former smoker | 15(34.09%) | 15(33.33%) |  |
| Current smoker | 18(40.91%) | 22(48.89%) |  |
| Years of diagnosis COPD | | | |
| <1year | 6(13.64%) | 5(11.11%) | 0.881^b^ |
| 1-5years | 7(15.91%) | 9(20.00%) |  |
| 5-10years | 22(50.00%) | 24(53.33%) |  |
| >10years | 9(20.45%) | 7(15.56%) |  |
| GOLD classification | | | |
| Ⅰ(mild) | 8(18.18%) | 6(13.33%) | 0.384^b^ |
| Ⅱ(moderate) | 18(40.91%) | 25(55.56%) |  |
| Ⅲ(severe) | 18(40.91%) | 14(31.11%) |  |
| Comorbidities | | | |
| Heart disease | 25(56.81%) | 22(48.89%) | 0.503^b^ |
| Diabetes | 7(15.91%) | 12(26.67%) |  |
| Hypertension | 6(13.64%) | 6(13.33%) |  |
| Others | 6(13.64%) | 5(11.11%) |  |

## Continuous values are presented as (mean±standard) deviation and categorical variables are presented as number (percentage).

## Legend: BMI=body mass index;Kg=kilograms;GOLD=Global initiative for chronic obstructive lung disease.

## ^a^ Independent t test. ^b^ Chi-squared test.

**Table 3**

Comparison of the outcome variables between the two groups before and after the intervention.

| **Variables** | **Group** | **Baseline(T0)** | **4weeks (T1)** | **8weeks (T2)** | **12weeks (T3)** | ***F*1(*p*)** | $\boldsymbol{\eta}^{\boldsymbol{2}}$ | ***F*2(*p*)** | $\boldsymbol{\eta}^{\boldsymbol{2}}$ | ***F*3(*p*)** | $\boldsymbol{\eta}^{\boldsymbol{2}}$ |
| --- | --- | --- | --- | --- | --- | --- | --- | --- | --- | --- | --- |
| PA | Exp | 1150.02±434.79 | 1300.40±387.17 ^a^ | 1350.67±347.34 ^a^ | 1408.44±361.78 ^a^ | 4.433  (**0.038**) | 0.048 | 5.155  (**0.003**) | 0.154 | 1.886  (0.138) | 0.062 |
|  | Con | 1162.50±420.87 | 1247.45±485.85 | 1164.20±387.17 | 1146.32±321.55 |  |  |  |  |  |  |
|  | t | 0.138 | -0.573 | -2.393 | -3.610 |  |  |  |  |  |  |
|  | p | 0.891 | 0.568 | **0.019** | **0.001** |  |  |  |  |  |  |
| SB | Exp | 7.24±0.92 | 6.47±0.83 ^a^ | 6.22±0.54^ab^ | 6.19±0.52 ^abc^ | 10.714  (**0.002**) | 0.110 | 23.030  (**<0.001**) | 0.448 | 14.817(**<0.001**) | 0.343 |
|  | Con | 7.23±0.10 | 7.04±0.96 | 6.75±0.72 | 7.03±0.90 |  |  |  |  |  |  |
|  | t | -0.052 | 2.980 | 3.872 | 5.356 |  |  |  |  |  |  |
|  | p | 0.959 | **0.004** | **<0.001** | **<0.001** |  |  |  |  |  |  |
| mMRC | Exp | 2.44±1.06 | 2.04±1.15 | 1.76±0.91 ^a^ | 1.67±0.91 ^ac^ | 6.893  (**0.010**) | 0.073 | 7.899  (**<0.001**) | 0.83 | 1.874  (0.138) | 0.021 |
|  | Con | 2.61±1.10 | 2.32±0.98 ^a^ | 2.34±1.08 ^a^ | 2.32±1.03 |  |  |  |  |  |  |
|  | t | 1.135 | 1.521 | 3.430 | 3.442 |  |  |  |  |  |  |
|  | p | 0.259 | 0.132 | **0.001** | **0.001** |  |  |  |  |  |  |
| Exercise self-efficacy | Exp | 52.96±5.42 | 55.29±5.43 ^a^ | 56.31±5.45 ^a^ | 63.31±8.10 ^abc^ | 22.282  (**<0.001**) | 0.204 | 10.840  (**<0.001**) | 0.277 | 17.274  (**<0.001**) | 0.379 |
|  | Con | 53.16±4.76 | 54.57±4.26 ^a^ | 55.25±5.41 ^a^ | 52.07±4.52 ^bc^ |  |  |  |  |  |  |
|  | t | 0.188 | -0.703 | -0.922 | 0.359 |  |  |  |  |  |  |
|  | p | 0.851 | 0.484 | 0.359 | **<0.001** |  |  |  |  |  |  |
| CAT | Exp | 16.62±2.75 | 15.04±2.02 ^a^ | 13.53±1.55 ^ab^ | 13.20±1.91 ^ab^ | 29.976  (**<0.001**) | 0.256 | 20.336  (**<0.001**) | 0.418 | 4.441  (**<0.001**) | 0.136 |
|  | Con | 17.11±2.79 | 16.02±2.14 ^a^ | 16.41±2.18 | 15.80±1.96 ^ac^ |  |  |  |  |  |  |
|  | t | 0.837 | 2.217 | 7.188 | 6.321 |  |  |  |  |  |  |
|  | p | 0.405 | **0.029** | **<0.001** | **<0.001** |  |  |  |  |  |  |
| 6MWD | Exp | 317.91±15.10 | 327.27±15.04 ^a^ | 324.47±12.05 ^ab^ | 336.13±11.98 ^ab^ | 13.465  (**<0.001**) | 0.134 | 14.875  (**<0.001**) | 0.344 | 30.545  (**<0.001**) | 0.519 |
|  | Con | 323.52±14.89 | 328.18±10.68 ^a^ | 318.18±110.68 ^ab^ | 311.48±11.41 ^abc^ |  |  |  |  |  |  |
|  | t | 1.765 | 0.457 | -6.743 | -9.938 |  |  |  |  |  |  |
|  | p | 0.081 | 0.649 | **<0.001** | **<0.001** |  |  |  |  |  |  |

## Legend：Significant valuesare in [bold].

## Exp=Experimental group;Con=Control group;PA=physical activity;SB=sedentary behaviour;mMRC=modified Medical Research Council dyspnoea scale;6MWD=6 minutes walking distance; CAT=COPD Assessment Test;*F*1=Group effect;*F*2=Time effect;*F*3=Interation effect.

## ^a^ Compared with baseline *P*<0.05.^b^ Compared with 4 weeks after treatment *P*<0.05.^c^ Compared with 8 weeks after treatment *P*<0.05.

**Table 4**

Difference-in-differences of lung function.

| Variables | Experimental group (*n*=45) | | | Control group (*n*=44) | | | DID (95% CI) | *P* value |
| --- | --- | --- | --- | --- | --- | --- | --- | --- |
|  | Baseline | 12 weeks | Within-group  effect (*P*) | Baseline | 12 weeks | Within-group effect (*P*) |  |  |
| FEV1% | 57.18±15.87 | 60.58±15.31 | 0.01 | 57.84±16.62 | 56.77±14.30 | 0.12 | -4.45 (-6.62 to -2.28) | **<0.001** |
| FEV1 | 1.61±0.35 | 1.69±0.34 | 0.23 | 1.58±0.60 | 1.59±0.34 | 0.96 | -0.11 (-0.34 to 0.13) | 0.37 |
| FVC | 2.78±0.56 | 2.76±0.58 | 0.37 | 2.67±0.92 | 2.76±0.55 | 0.61 | -0.07 (-0.46 to 0.33) | 0.74 |
| FEV1/FVC | 58.03±6.94 | 59.12±6.75 | 0.13 | 58.98±6.71 | 57.88±6.94 | 0.43 | -2.40 (-6.14 to 1.33) | 0.21 |

## Legend: FEV1=forced expiratory volume in 1 s; FVC=forced vital capacity; DID=Difference-in-difference.

## **Fig 1.**

## Flowchart of the intervention for the protocol.

##
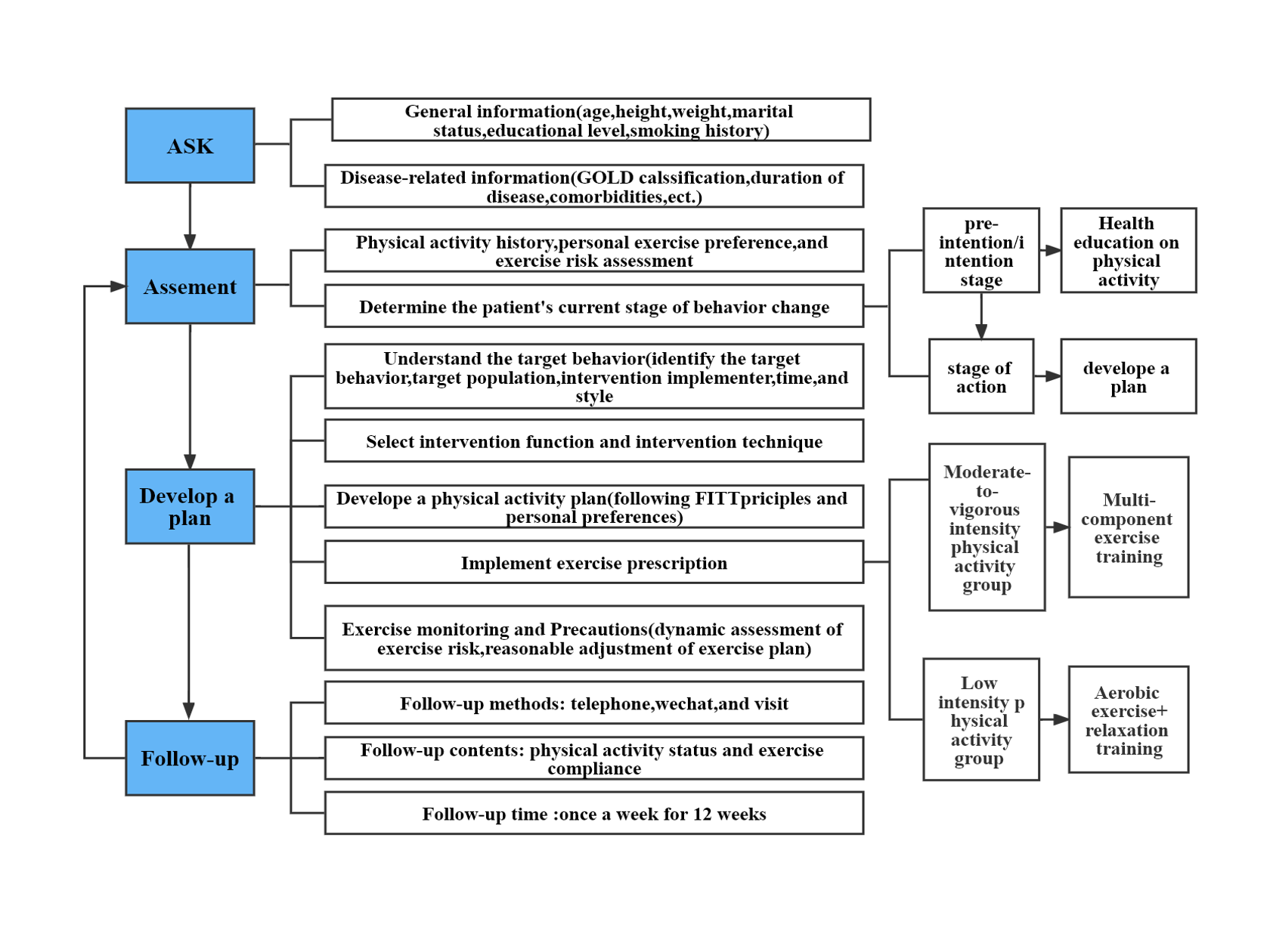


## **Fig 2.**

Study flowchart.

## **
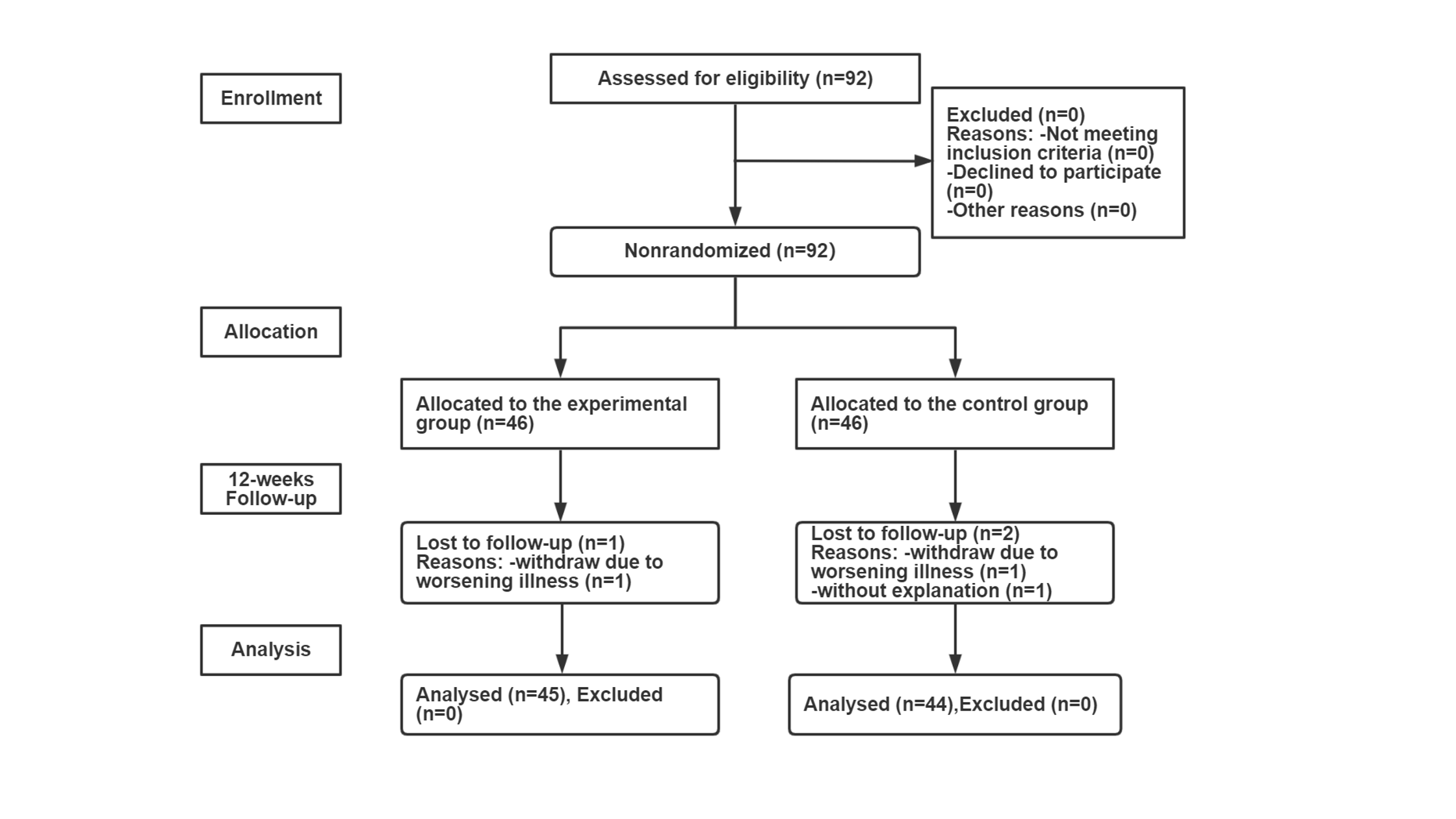
**

## **Fig 3.**

Changes in outcomes (a) total physical activity, (b) Sedentary time(c), mMRC, (d) 6MWD, (e) Exercise self-efficacy, and (f) CAT over time in the experimental and control group.
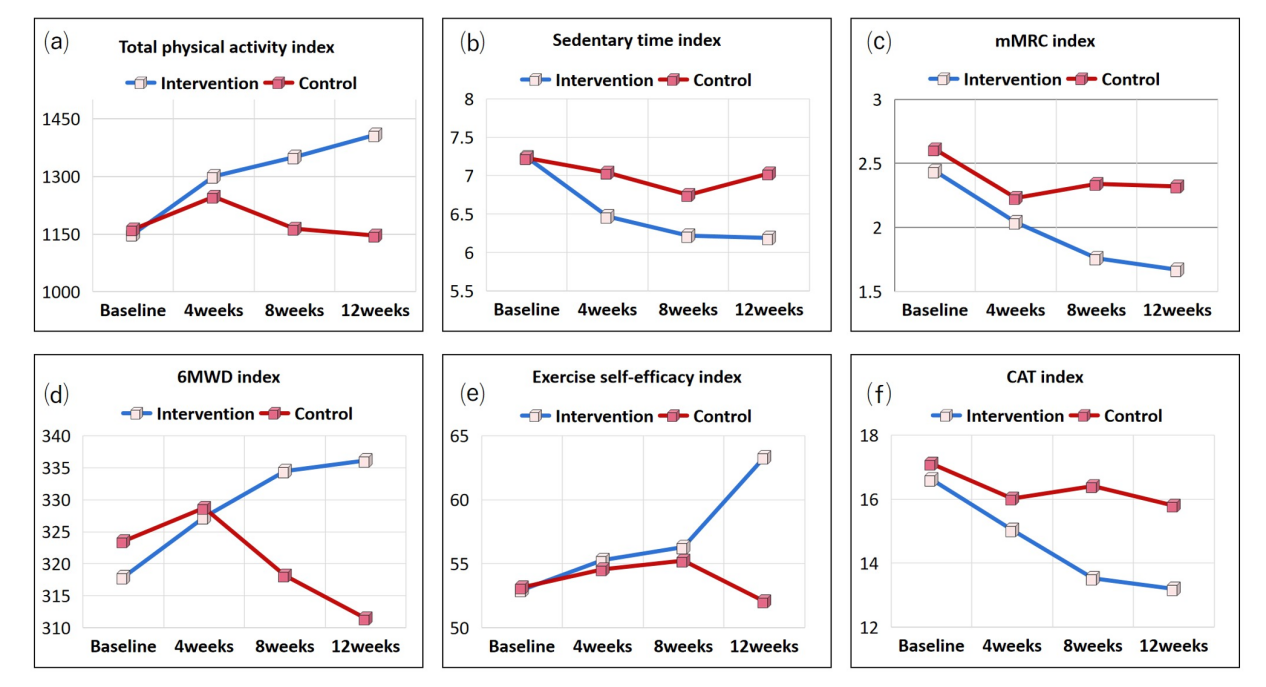

Supplement: Supplementary file 1 — Supplementary Information 1. [file 41598_2023_50099_MOESM1_ESM.docx]
