# Supplementary material for: Development of a behavior change intervention to improve physical activity in patients with COPD using the behavior change wheel: a non-randomized trial
Source: Sci Rep. 2023 Dec 21;13:22929. doi: 10.1038/s41598-023-50099-z (PMC10739839; doi:10.1038/s41598-023-50099-z)
Supplement: Supplementary file 2 — Supplementary Information 2. [file 41598_2023_50099_MOESM2_ESM.docx]

**Additional file 2.** Development phase for content (Behaviour Change Wheel)

The Behaviour Change Wheel (BCW) process of intervention development involves three key stages: (1) understanding the behavior, (2) identifying intervention functions, and (3) identifying content and the intervention options.

Stage 1: understanding the behavior

| Target behavior | Improve physical activity levels and reduce sedentary time in people with COPD |
| --- | --- |
| Who will they do it? | People with COPD |
| When will they do it? | When convenient to the people with COPD |
| Where will they do it? | At home or outdoors |
| How often will they do it? | 150 minutes walking/week for 12 weeks(30 minutes/day, 5 days/week) |
| With whom will they do it? | Alone or with family |

Stage 2: identifying intervention functions

Mapping of the physical activity Intervention Components to the COM-B Model and the Selected Intervention Functions.

| Intervention Component | BCW Intervention Functions | COM-B Constructs |
| --- | --- | --- |
| Assess physical activity levels | Education  Persuasion  Environmental restructuring  Enablement | Capability—physical  Opportunity—physical and social  Motivation—reflective |
| Develop a physical activity plan | Education  Training  Enablement | Capability—physical and psychological  Opportunity—physical and social |
| Goal setting | Persuasion  Incentivisation  Enablement | Motivation—reflective  Opportunity—physical and social |
| Problem solving | Enablement | Opportunity—physical  Capability—psychological |
| Health education | Education  Persuasion  Training | Capability—physical and psychological  Opportunity—physical and social |
| Exercise Instruction Video | Education  Training | Capability—physical  Opportunity—physical |
| Self-monitoring heart rate | Training | Capability—physical |
| Pedometer | Environmental restructuring  Enablement | Capability—psychological  Motivation—reflective and automatic |
| Reminder card | Environmental restructuring  Enablement | Opportunity—physical |
| Physical activity logbook | Training  Enablement | Capability—psychological  Motivation—automatic |
| Exercise consultation | Persuasion | Motivation—reflective and automatic |
| Social support | Environmental restructuring  Enablement | Opportunity—social |
| Set an example | Modeling | Motivation—reflective and automatic |
| Feedback on behaviour | Education  Persuasion  Enablement | Motivation—reflective and automatic |

Stage 3: identifying content and the intervention options

## Based on stage 1 and 2, 19 behavioral change strategies to implement within physical activity programmes were identified.

| COM-B behavioral  determinants | | Intervention component | Intervention functions | BCTs* |
| --- | --- | --- | --- | --- |
| Motivation | Reflective motivation | The concept of exercise rehabilitation was introduced in detail, and the importance of physical activity for disease prognosis was emphasized. Regular physical activity is beneficial to physical and mental health. Correct patients' wrong cognition, establish a correct concept of the disease, and stimulate patients' reflective motivation | Education  Persuasion  Training | 1.1Goal setting (behaviour)  1.2Problem solving  1.3Goal setting (outcome)  2.2Feedback on behaviour  2.7Feedback on outcome(s) of behaviour  5.1Information about health consequences |
|  |  | Through motivational interview, the current behavior change stage of patients was determined: if the pre-intention/intention stage, the health education of physical activity knowledge should be strengthened; In the preparation/action stage, patients should be helped to develop an action plan and relevant information support should be provided |  |  |
|  |  | Assess the patient's physical activity level regularly, including the type, intensity, time and frequency of activity, and provide feedback to the patient |  |  |
|  | Automatic motivation | Make patients aware of the importance of rehabilitation exercise and cultivate their exercise habits | Education  Enablement  Training | 2.3Self-monitoring of behaviour  8.3Habit formation |
|  |  | Set exercise goals, develop exercise plans and coping strategies |  |  |
|  |  | Help patients to arrange their activity time reasonably, recommend exercise intensity, and enhance patients' exercise self-efficacy |  |  |
| Capability | Psychological capability | Provide health information about physical activity：  Face-to-face interviews or health brochures can be used during hospitalization to inform patients about the health benefits of physical activity and the dangers of prolonged sitting; After discharge, wechat or telephone follow-up can be used to continuously strengthen the risk cognition of patients | Education  Enablement  Training | 5.1Information about health consequences  9.3Comparative imagining of future outcomes |
|  | Physical capability | Provide exercise-related matters for attention, push videos about exercise, improve exercise skills and reduce exercise risks for patients | Training  Enablement | 1.1Goal setting (behavior)  1.4Action planning  1.6Discrepancy between current behavior and goal  2.3Self-monitoring of behaviour  4.1Instruction on how to perform the behavior |
|  |  | Assess the physical activity level of patients, make exercise plans according to the needs of patients, and follow the exercise principle of step by step and according to one's ability |  |  |
|  |  | Outdoor activities, such as walking, jogging, etc., should choose smooth and spacious roads, appropriate shoes and socks, pay attention to keep warm and avoid cold |  |  |
| Opportunity | Physical opportunity | Set a reminder card or alarm clock to remind you of sitting for long periods of time. Get up and move for 1 to 2 minutes every 30 minutes | Environment restructuring  Training  Restriction | 1.2Problem solving  7.1Prompts/cues  12.1Restructuring the physical environment |
|  |  | Provide a daily activity log sheet to guide patients to record their daily activities and experiences |  |  |
|  |  | Put a pair of sneakers in front of your home as a reminder |  |  |
|  |  | Use a wechat pedometer to monitor your daily steps |  |  |
|  |  | Provide exercise equipment such as elastic bands to patients and push exercise instruction videos |  |  |
|  | Social opportunity | Provide social resources (support from family, friends and health care professionals)  During hospitalization, nurses should timely understand the needs of patients, persuade patients to cooperate with treatment and nursing, and verbal encouragement, to help patients establish confidence in exercise | Modeling  Enablement | 3.2Social support (practical)  3.3Social support (emotional)  5.4Monitoring of emotional consequences 6.1Demonstration of the behaviour 11.2Reduce negative emotions |
|  |  | Encourage patients' family members to participate in the activities and improve patients' enthusiasm for sports |  |  |
|  |  | After discharge, patients can contact medical staff through wechat and telephone at any time for communication and consultation |  |  |

BCTs*, behavior change technique

Michie S, Richardson M, Johnston M, et al. The behavior change technique taxonomy (v1) of 93 hierarchically clustered techniques: building an international consensus for the reporting of behavior change interventions. Ann Behav Med. 2013;46(1):81-95.
